# Interaction of pulsed low frequency electromagnetic field (PEMF) with mitochondria

**DOI:** 10.1038/s41598-026-37527-6

**Published:** 2026-01-30

**Authors:** Sergejs Zavadskis, Andreas Sebastian Gasser, Miriam Karas, Sara Kostrebic, Jonas Flatscher, Annette Vaglio-Garro, Peter Dungel, Heinz Redl, Johannes Grillari, Adelheid Weidinger, Paul Slezak, Andrey V. Kozlov

**Affiliations:** 1 Ludwig Boltzmann Institute for Traumatology, The Research Center in Cooperation with AUVA, Austrian Cluster for Tissue Regeneration, Vienna, Austria; 2 Institute for Molecular Biotechnology, BOKU University, Vienna, Austria

**Keywords:** Biochemistry, Biophysics, Cell biology, Physiology

## Abstract

Pulsed electromagnetic field (PEMF) therapy is a non-invasive treatment that delivers electric and magnetic fields to tissues via inductive coils inducing salutary effects. Previously PEMF was reported to accelerate gas transport in the liquid phase. Drawing analogies from electrical elements, we hypothesized that PEMF can modulate transmembrane potential of biological membranes. Given that mitochondria interact with gaseous molecules such as oxygen and nitric oxide (NO) and contain voltage-sensitive channels, this study focused on the effects of a single PEMF device with a low input-energy and a 1ms, 30 kHz sine wave duty cycle per pulse on mitochondrial function. Experiments were conducted using cell cultures, tissue homogenates, and isolated mitochondria. We assessed mitochondrial membrane potential, NO levels, and mitochondrial respiration. We found no evidence that PEMF restores mitochondrial respiration inhibited by NO, but it can be restored by exposure of mitochondria to blue light. Our findings show that PEMF selectively stimulates respiration linked to ATP-synthesis affecting less uncoupled respiration. This suggests that changes in ATP-synthesis underlies the primary beneficial effects observed here. The underlying mechanisms may include interaction of PEMF with mitochondrial transport systems or activity of mitochondrial complexes. The exact mechanisms still should be investigated.

**Supplementary Information:**

The online version contains supplementary material available at 10.1038/s41598-026-37527-6.

## Introduction

Pulsed electromagnetic field (PEMF, also attributed to low-frequency electromagnetic field (LF-EMF)) therapy is a non-invasive treatment method that delivers electric and magnetic fields to tissues via inductive coils. A growing body of clinical evidence supports its use in both animals and humans for specific indications, including bone healing, wound healing, osteoarthritis, inflammation, and the management of post-operative pain and edema^1–5^.

PEMF devices are typically made up of a resonant circuit with a capacitor connected to a coil looped inductor via a semiconductor switch. The capacitor is pre-charged before discharging its load into the inductor to initiate oscillations of the resonant circuit. The resonant circuit then oscillates until losses dissipate the energy. A new technical approach as used in this work (Hofmeir Magnetics Limited) employs a parallel resonant circuit in which the semiconductor switch is excluded from the oscillating pathway. This design extends pulse duration to ~ 1 ms, lowers operating voltages to 150–350 V, and reduces currents to 100–1500 resulting in stable, sinusoidal pulses. Of note, all PEMF devices have a fixed EMF frequency which can be different from one PEMF device to another suggesting that theoretically effects observed with one device can be different or not occur with other devices. That is the major problem rising by the interpretation of biological effects induced by PEMF - treatments.

Although numerous biological effects of PEMF have been reported, its primary target within biological systems remains poorly understood due to limited knowledge about underlying mechanisms as well as due to low reproducibility of original experimental conditions^6^. Many of the observed benefits of PEMF are believed to improve energy metabolism^7–9^, though the exact mechanisms underlying these metabolic improvements are still unclear.

Theoretically PEMF as any other treatment, can have deleterious, beneficial or no effect. The first aim of this study was to test whether it has any effect on mitochondrial function in intact cells. We exposed cells to PEMF and determined parameters relevant for mitochondrial function including the respiration rate, the levels of reactive oxygen and nitrogen species, which are often associated with the deleterious action of mitochondria. Later we focused more on known mechanism of PEMF and biologically relevant systems.

One of the most known effects of EMF is its interaction with cavitation namely behaviour of gas bubbles in liquids, covering their formation, motion, shape changes, and collapse. In addition to lipid–water systems, EMF can significantly influence gas–water interfaces, due to charge accumulation at the boundary between the two phases^10^. These interfacial charges can modulate local gas dependent processes. For instance, EMF have been shown to alter the movement of oxygen microbubbles, thereby facilitating reactions, in which oxygen is a key reactant^11^. Another study reports that low-frequency EMF treatment of water has an outgassing effect, enhancing the vaporization of dissolved carbon dioxide^12^.

These reports again implicate mitochondria as potential (P)EMF targets, given that mitochondrial respiration is highly dependent on the diffusion of oxygen to cytochrome c oxidase (complex IV). Additionally, gases such as NO modulate mitochondrial function under pathological conditions. During inflammation, elevated NO levels can inhibit complex IV; under such conditions, promoting NO clearance (e.g., through enhanced evaporation) may support mitochondrial recovery.

Therefore, the second aim of this study was to investigate how PEMF modulate intact mitochondrial function and mitochondria interaction with NO.

Biological membranes can be another target of PEMF. It has been proposed that biological structures can be modelled as equivalent electrical circuits comprising resistive and capacitive elements^13^. Specifically, the phospholipid bilayer of the cell membranes functions as a dielectric insulator separating two conductive (electrolytic) water media, both together effectively acting as a capacitor^14^.

Together, membrane capacitance and resistance determine the membrane time constant, which theoretically defines how quickly the membrane potential can respond to ionic currents flowing through ion channels^15^. This behaviour parallels that of electrical AC circuits, which filter specific frequencies. The capacity of biological membranes is rather stable and was estimated as approx. 1 µF/cm². In contrast, the impedance of cytoplasm membranes is varying at different conditions in a range of 1 kiloohm to 20 megaohm^16^. Nordin et al. showed that the impedance of cell monolayer substantially drops upon exposure to increased frequencies of electromagnetic field^17^. Moreover, in a single bilayer membrane, the impedance was as low as 30 ohm^18^. Such variation of impedance in biological membranes and in cellular structures suggests that biological objects can be susceptible to a wide range of electromagnetic field (EMF) frequencies.

The electric field component of an external electromagnetic wave can directly charge or discharge this “capacitor” — a process known as dielectric polarization — generating an internal electric field within the membrane that opposes the external field^10^. This interaction can alter the transmembrane potential. By modulating membrane potential, PEMF may regulate biological processes governed by voltage-dependent mechanisms, such as the activity of voltage-gated ion channels. Indeed, there is growing evidence that electromagnetic fields can influence membrane permeability^4^.

In this context, mitochondrial membranes may serve as primary targets for EMF, since the most abundant protein in the outer mitochondrial membrane (OMM) is Voltage-Dependent Anion Channel (VDAC)^19,20^. VDAC facilitates the transport of low-molecular-weight mitochondrial substrates and adenosine phosphates, playing a central role in mitochondrial metabolism and cross-membrane exchange. To access these processes, we compared effect of PEMF on uncoupled respiration, characterising the efficiency of mitochondrial electron transport chain (ETC) and coupled respiration characterising efficiency and adenosine diphosphate (ADP) phosphorylation.

Thus, the effect of PEMF on coupled and uncoupled respiration was the third aim of this study.

The study was carried out in cell culture, tissue homogenate, and isolated mitochondria. As a PEMF source a device with a long duty cycle paired with a low input energy was used to provide a sinus shaped PEMF signal.

## Results

### Effect of PEMF on the mitochondrial membrane potential, intracellular levels of nitric oxide, reactive oxygen species and mitochondrial respiration

**Fig. 1:**
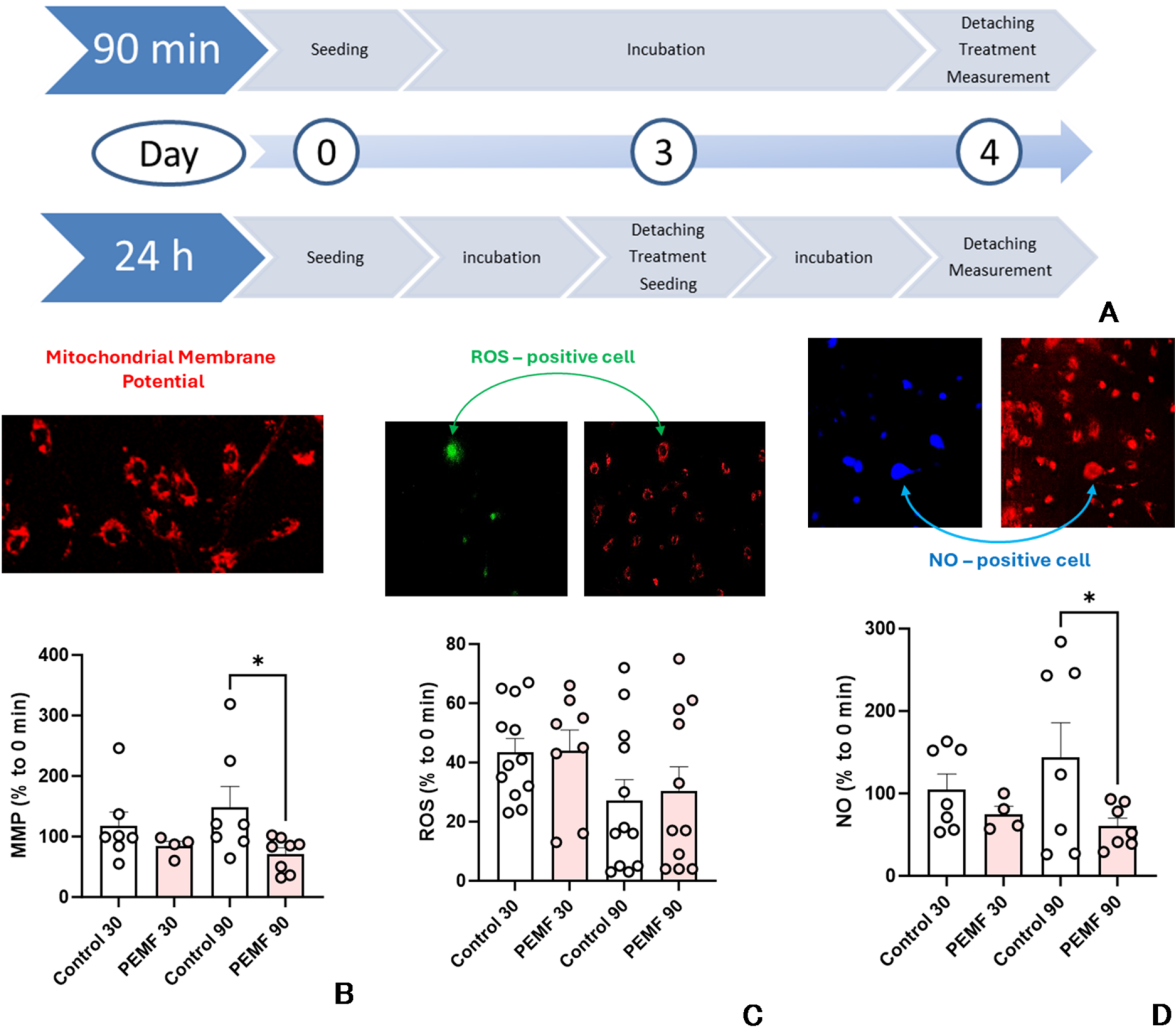
Experimental protocol (**A**) and determination of membrane potential (**B**), reactive oxygen species (**C**) and nitric oxide (**D**) levels in non-permeabilized LHCN-M2 cells. Statistics: The data were analyzed by two tailed t-test vs. corresponding controls; experimental points (n=) are shown in the corresponding bars, the data are presented as mean ± SEM (error bars), * - *p* < 0.05. MMP – mitochondrial membrane potential; ROS – reactive oxygen species; NO – nitric oxide. Conclusion: We observed a decrease in MMP and NO levels 90 min after PEMF treatment. No changes in ROS levels were found.

Figure 1 shows results on the influence of PEMF on the mitochondrial membrane potential (MMP), reactive oxygen species (ROS), and nitric oxide (NO) in cell culture. The experimental protocol is shown on Fig. 1A. We investigated the effects of PEMF 30 min and 90 min after exposure (see methods section for details). In addition, we investigated respiratory function of mitochondria in permeabilized cells 90 min and 24 h after PEMF treatment (Fig. 1A h).

**Fig. 2:**
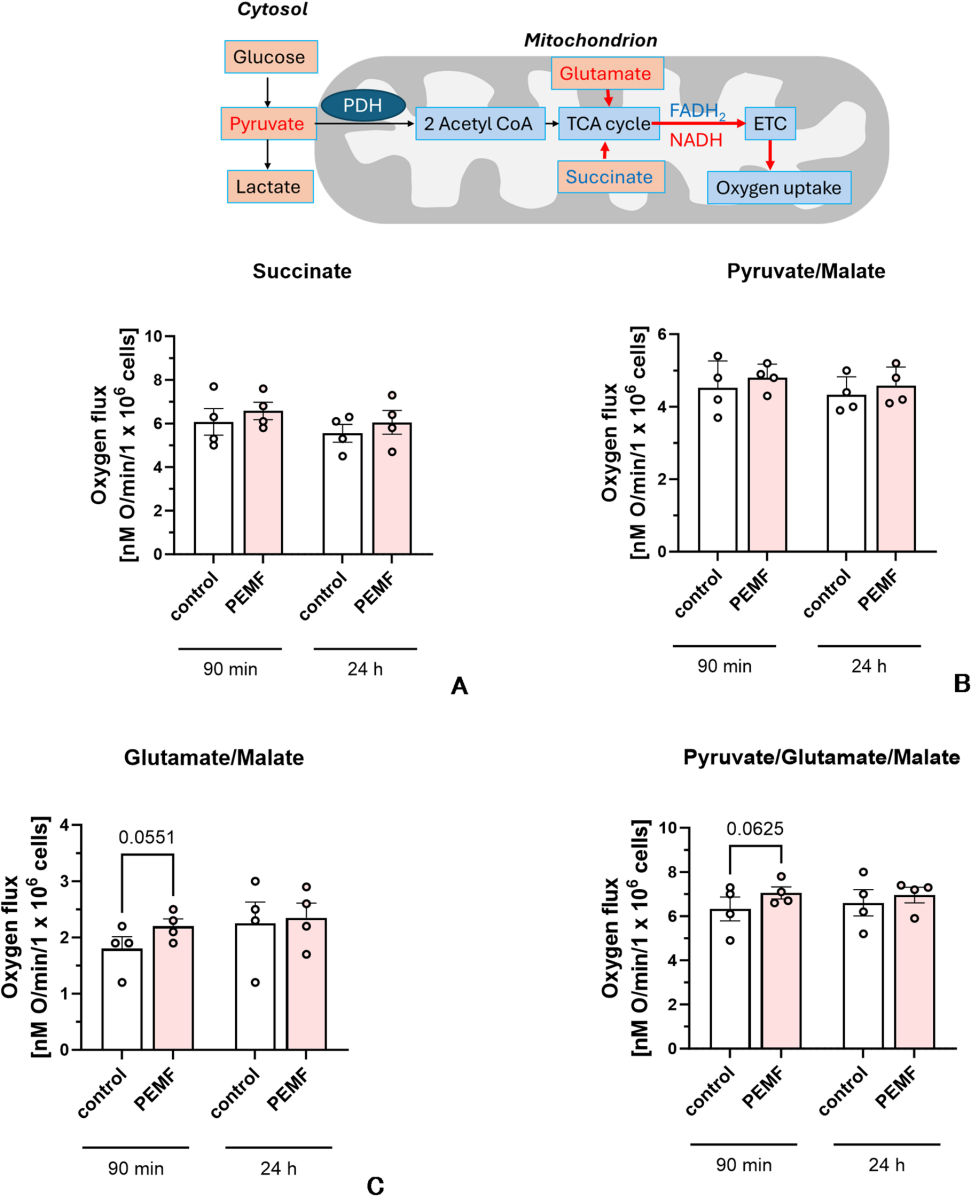
Effect of PEMF on the mitochondrial function (state 3 respiration) in permeabilized cells with different mitochondrial substrates. Inset on the top illustrates pathways of electron supply to ETC from different mitochondrial substrates, (**A**) Mitochondrial respiration in the presence of substrate of complex II, succinate. (**B**) Mitochondrial respiration in the presence of substrate of complex I, pyruvate. (**C**) Mitochondrial respiration in the presence of glutamate. (**D**) Mitochondrial respiration in the presence of glutamate, malate and pyruvate. Conclusion: Here we have observed a substantial but not significant increase in mitochondrial respiration linked to ATP synthesis by 90 min after PEMF treatment in the presence of glutamate. Succ – succinate; G – glutamate; M – malate; P – Pyruvate. Statistics: The data were analyzed by two tailed t-test vs. corresponding controls; experimental points (n=) are shown in the corresponding bars, the data are presented as mean ± SEM (error bars), *p < 0.05.

MMP significantly decreased after 90 min of PEMF treatment. A decrease in MMP can be due to several reasons, including increased permeability of the inner mitochondrial membrane, dysfunction of the mitochondrial respiratory chain, or elevated ATP synthesis. We did not observe any significant changes in intracellular ROS generation in response to PEMF, but a reduction in NO levels following PEMF treatment. Given that NO is a known inhibitor of mitochondrial respiration and ATP synthesis, we hypothesize that PEMF may induce the dissociation of NO from complex IV and facilitate mitochondrial respiration. Elevated respiration may drop the inner mitochondrial membrane (IMM) potential. To test this hypothesis, we performed experiments on cells with a permeabilized cytoplasm membrane and examined their mitochondrial respiration. Permeabilization was necessary to allow mitochondrial substrates and adenosine diphosphate to diffuse from incubation media to the mitochondria. The data are presented in Fig. 2.

In these experiments we have observed that the mean value of respiration was always higher in the group treated with PEMF and containing glutamate, but the increase did not reach significance. Considering the similarity of the PEMF effects on mitochondrial respiration in the presence of glutamate along with other substrates we additionally analyzed data sets from all samples containing glutamate with 2-way ANOVA. This analysis shows that there is significant interaction between mitochondrial respiration and both PEMF exposure and substrate composition (Table 1). We did not observe any deleterious effect of PEMF.

**Table 1:** Two-way ANOVA analysis of the effects of PEMF on the mitochondrial respiration. The G and PGM groups (see Fig. 2) were analysed.

Source of variation	% of total variation	*P* value	*P* value summary	Significant?
Effect of PEMF treatment	1.341	0.0260	*	Yes
Effect of Substrates	93.12	< 0.0001	****	Yes

### The impact of PEMF and NO on mitochondrial respiration

**Fig. 3:**
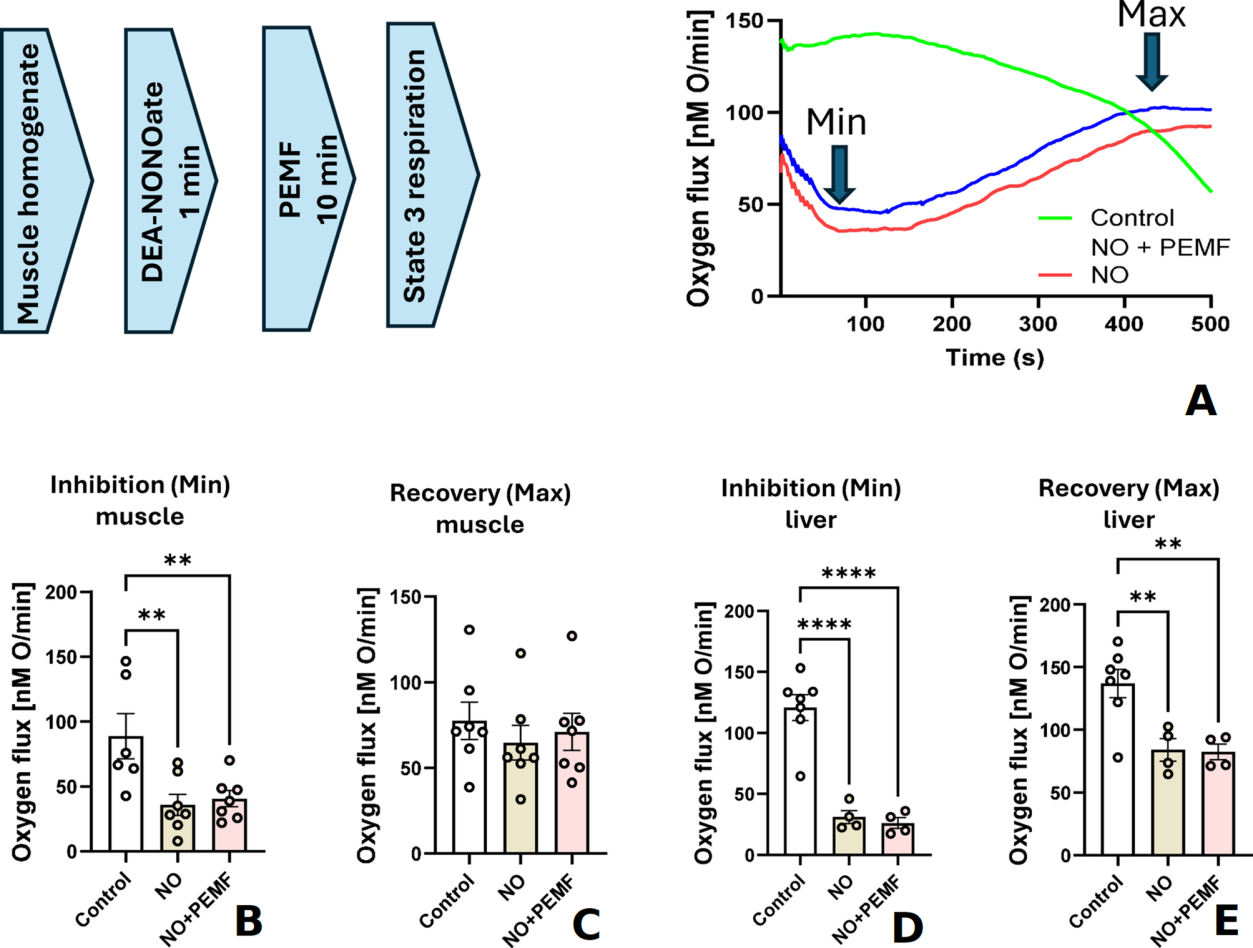
Effect of NO and PEMF on the respiration activity of mitochondria. (**A**) Experimental design and a typical experiment with muscle homogenates treated with an NO-donor, releasing 25 µM of NO over 10 min. (**B**, **C**) Effect of NO and PEMF in muscle homogenate. (**D**, **E**) Effect of NO and PEMF in liver homogenate. State 3 mitochondrial respiration was stimulated by 10 mM glutamate/malate and 2 mM ADP. During the recovery of State 3 respiration, we determined the minimal slope (full inhibition by NO) and maximal slope (highest recovery rate). Conclusion: The data suggests that PEMF does not facilitate recovery following NO-induced inhibition of liver mitochondria either in muscle or in liver homogenates. PEMF – pulse electromagnetic field; NO – nitric oxide. Statistics: The data were analyzed by one-way ANOVA followed by Holm-Sidak’s multiple comparisons test. The data are presented as mean ± SEM (error bars), experimental points (n=) are shown in the corresponding bars. ** - p < =0.01; **** *p* < 0.0001.

We hypothesized that this interaction between PEMF and mitochondrial respiration is mediated by the substitution of NO by oxygen at complex IV resulting in decrease of NO levels which we observed in previous experiments (see Fig. 1). To test this hypothesis, we exposed muscle and liver homogenates to exogenous NO and after mitochondrial respiration was inhibited, we treated the homogenates with PEMF. This experiment simulates elevated NO levels occurring upon inflammatory conditions. As an NO source we used the NO donor DEA-NONOate. Since, we could not exclude that PEMF can facilitate the release on NO from the NO Donor, we first performed preliminary experiments investigating the effect of PEMF on the NO release from the DEA-NONOate. To determine the NO release kinetics the reaction chamber of nitric oxide analyzer (NOA) was placed in the PEMF loop. The experimental design (Suppl. Figure 1) and the obtained data (Suppl. Figure 2, 3, 4) are shown in supplemental materials. In these experiments we tested different incubation temperatures and concentrations of DEA-NONOate. In all these experiments we did not observe any effect of PEMF on the NO release from NO-donor. As expected, the NO release was increased with the increase in the temperature or the NONOate concentration (Suppl. Figure 2, 3, 4). The shapes of the NO release traces were similar at different NONOate concentrations. Next, we started experiments with tissue homogenates exposed to both the NO-donor and PEMF. The results of these experiments (presented in Fig. 3) show that under the experimental conditions PEMF does not reverse NO-mediated inhibition of mitochondrial respiration. The latter suggests that PEMF does not facilitate the dissociation of NO from complex IV. However, we could not exclude that this negative result may be due to the specificity of our experimental model. While homogenates provide conditions where mitochondria are in a more natural environment, the concentration of mitochondria in the homogenate is significantly lower than within cells. Perhaps, the fraction of mitochondria affected by PEMF was too small to be detected. Based on this consideration we next performed experiments in mitochondrial suspensions with a high concentration of mitochondria, which was even approx. 3 fold higher than in non-homogenized tissue. Since in muscle and liver homogenates we observe similar results, but they were more pronounced in the liver homogenate, we isolated mitochondria from liver.

### The impact of PEMF, NO, and blue light on the respiration of mitochondrial suspension

**Fig. 4:**
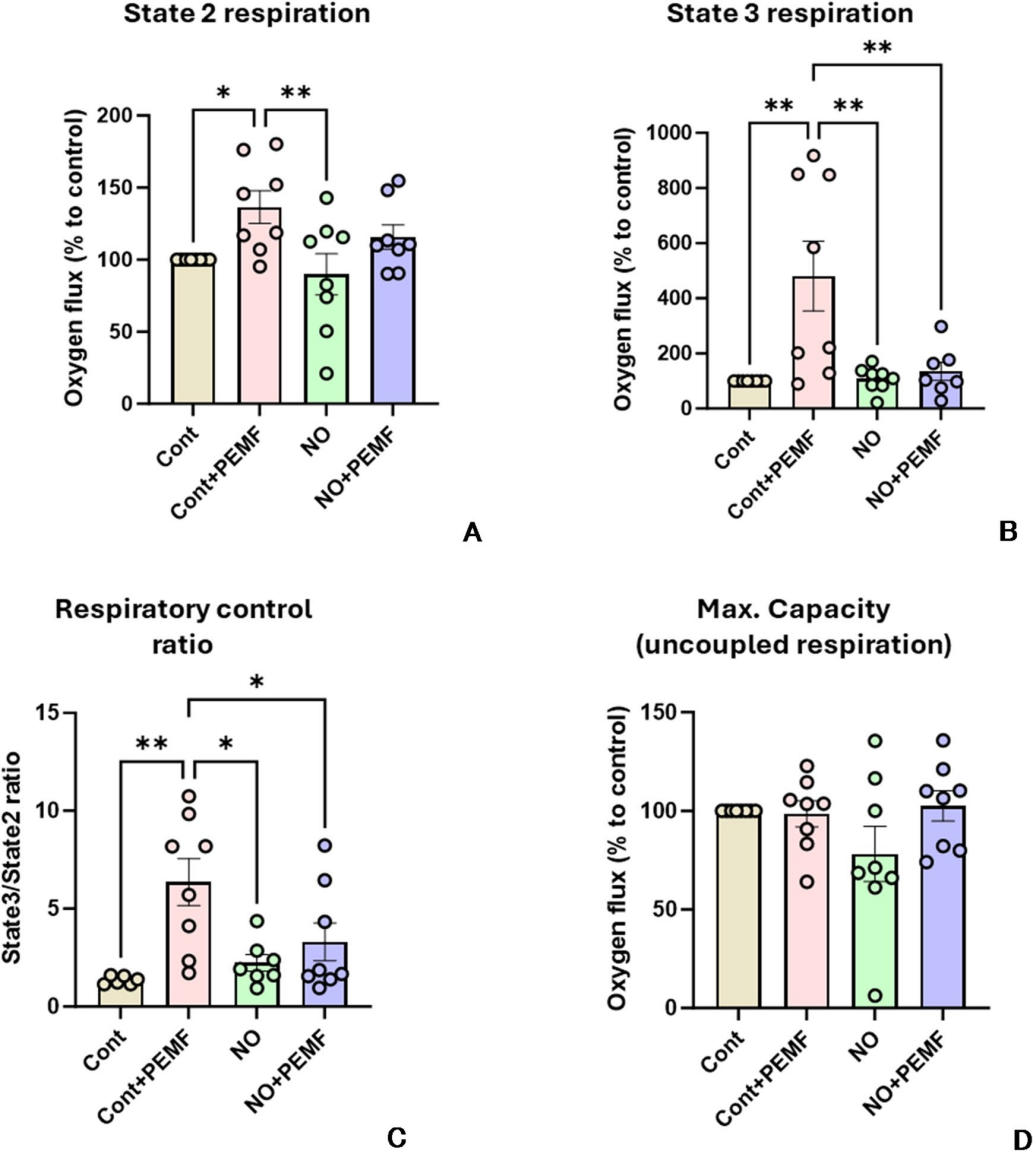
Effect of PEMF treatment on the mitochondrial function in control mitochondria and mitochondria inhibited by NO. We determined State 2 (**A**), state 3 (**B**), respiratory control (**C**) and total capacity (**D**). Statistics: Data are presented as mean+/- SEM. Statistical evaluation was performed by one-way ANOVA followed by Holms-Sidak post Hoc test. * - *p* < 0.05; ** - *p* < 0.01. Cont - control; PEMF – Pulse electromagnetic field; NO – nitric oxide. Conclusion: PEMF affects state 3 respiration, but not total mitochondrial capacity. PEMF does not facilitate recovery of mitochondria inhibited by NO.

In mitochondrial suspensions, similarly to all previous experiments, we did not observe any effect of PEMF on mitochondria inhibited by NO. The degree of inhibition was similar in mitochondria treated with DEA-NONOate irrespectively whether they were treated with PEMF or not. (Fig. 4). In contrast, in control mitochondria, we observed a slight but significant elevation of state 2 respiration and a strong increase in the state 3 respiration rate. In addition, the respiratory control ratio (state 3/state 2) characterizing mitochondrial coupling was also significantly higher. In contrast, we did not observe remarkable difference in the total capacity of mitochondrial respiration in the presence of uncoupler FCCP (Carbonylcyanid-p-Trifluormethoxyphenylhydrazone), so called uncoupled respiration reflecting electron flow through ETC. This finding was in line with the results in cell culture experiments described above. The absence of PEMF effect on mitochondria inhibited by NO can be generally due to 2 reasons, PEMF does not affect the process of ETC inhibition by NO, or the mitochondrial inhibition became irreversible and cannot recovered. To address this issue, we used previously described method to dissociate NO from complex IV by the blue light. Following this strategy, we tested the effect of blue light on control mitochondria and mitochondria treated by PEMF.

**Fig. 5:**
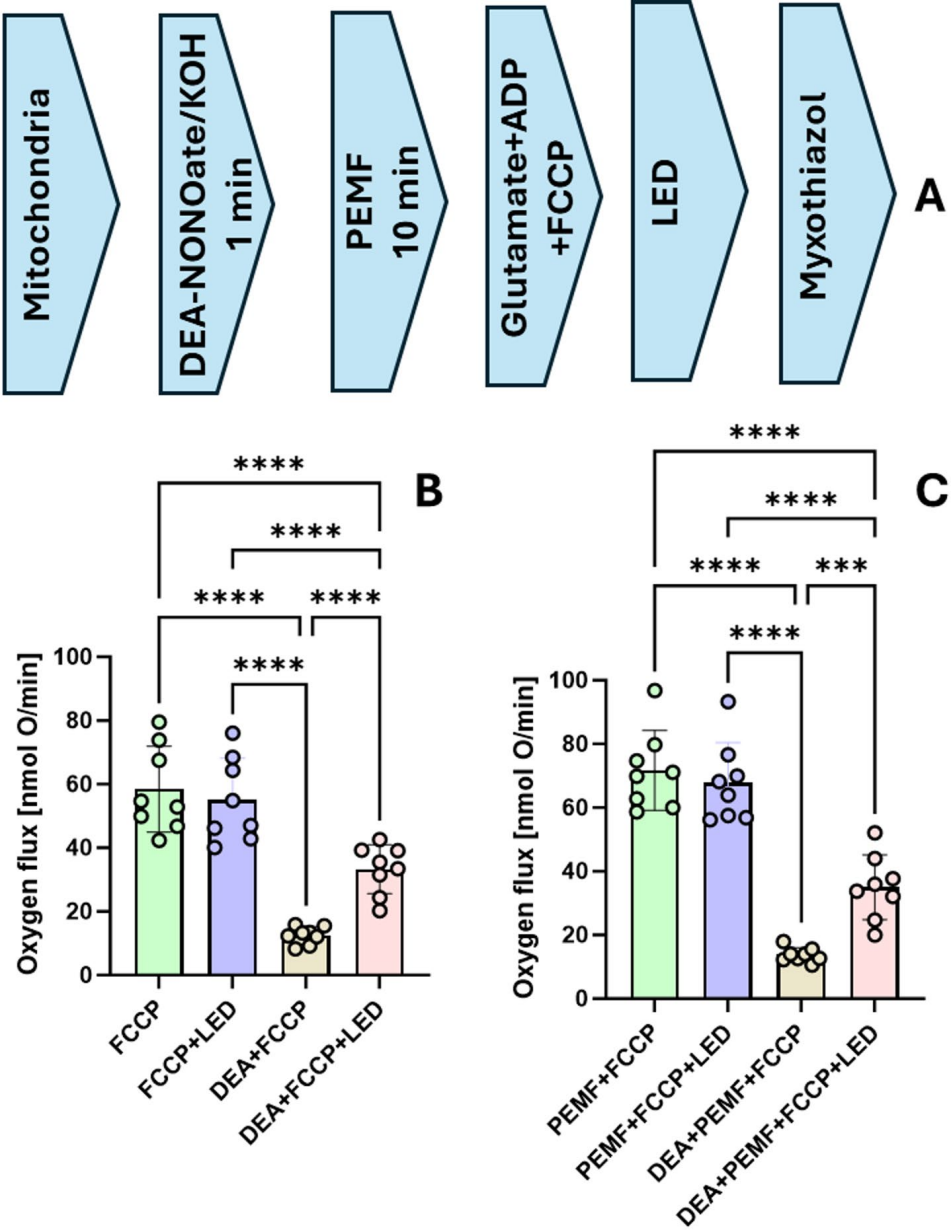
Combined effect of PEMF and blue light on control mitochondria and mitochondria inhibited by NO. (**A**) Experimental design of the experiment; (**B**) Effect of DEA NONOate on control mitochondria (**C**) effect of DEA NONOate on mitochondria treated. The samples were exposed to the blue light (470 nm) directly in the chamber of the high-resolution respirometer. Statistics: Data are presented as mean+/- SEM. Statistical evaluation was performed by one-way ANOVA followed by Holms-Sidak post Hoc test. * - *p* < 0.05. FCCP – Carbonyl cyanide p-trifluoromethoxyphenylhydrazone; DEA – DEA-NONOate – nitric oxide (NO) donor; LED – blue light source; PEMF – Pulse electromagnetic field. Conclusion: Blue light partially recovers total capacity of mitochondrial respiration (uncoupled respiration) There is no significant difference between effect of blue light on mitochondria treated and not treated by blue light. In this experiment the uncoupled respiration was slightly higher in mitochondria treated with PEMF (Mean±SEM (nmol O/min): 58,5±4,5 vs. 71,7±4,1 (*p* < 0.044).

The results of this experiment are shown in Fig. 5. We show that maximal capacity of mitochondrial ETC not treated with NO is not sensitive to blue light, the oxygen flux remained unchanged. In contrast, blue light partially recovers maximal capacity of mitochondria inhibited by NO. The recovery however was only partial, and we did not observe any difference between PEMF treated and not treated mitochondria. In contrast to previous experiments here we observed a slight increase in total capacity of mitochondria treated with PEMF. The latter may reflect slight changes in the experimental protocol (increased K+ levels and buffer capacity). This increase in total capacity can be attributed to beneficial effects of PEMF.

## Discussion

This study was focussed on the effect of PEMF on bioenergetic processes. The first aim was to test if it has any effect on mitochondrial function in intact cells. In these experiments we did not find any deleterious effect of PEMF. Of note, we did not observe any harmful effect of PEMF in the biological system we have studied. In contrast, we observe beneficial changes in mitochondrial respiration. Our attention was attracted by the fact, that we observed the changes in intracellular NO levels which indirectly suggested that the PEMF target interaction between NO and complex IV. As we described in introduction, from the background literature we learned that PEMF primarily affects the processes which occur on the border between different phases, such as gas-liquid borders, and facilitate either gas transport or gas evaporation.

Therefore, the next aim was to test whether PEMF can facilitate the dissociation of NO from complex IV, improving respiratory function of mitochondria, or not. This effect could be mediated by induction of cavitation by PEMF. We expected that PEMF could facilitate oxygen delivery, e.g. in a form of gas bubbles, to mitochondria. Elevated levels of oxygen in mitochondria expected to substitute NO by oxygen at complex IV. We tried to detect this phenomenon in three different models, cell, culture, tissue homogenates, and mitochondrial suspension, but we did not find it. In contrast to changes in the respiratory activity of mitochondria we did not find any evidence that PEMF can facilitate such gas exchange between the medium and respiratory complex IV of mitochondria. Theoretically we expected that PEMF could either facilitate oxygen delivery to mitochondria and increase both coupled and uncoupled respiration, but this was not the case. Considering that in this model NO can irreversibly damage mitochondria, making impossible their recovery we decided to use an independent method facilitating dissociation of NO from complex IV. Our previous studies showed that blue light can directly react with NO-complex IV complex, facilitate the release of NO and stimulate mitochondrial respiration^21^. Thus, we applied blue light to the samples treated with NO and PEMF and indeed we observe partial recovery of mitochondrial respiration. In this experiment we did not observe any difference in recovery rate between PEMF treated and not treated mitochondria. This suggests that the biological effects of light and PEMF are independent.

Next, we were trying to access the primary targets of PEMF in biological systems making an analogy between biological membranes and electrical elements. Since mitochondria contain voltage driven channels in their OMM we further focussed this study on the effect of PEMF on the mitochondrial function.

We have observed that PEMF stimulates mitochondrial respiration. This effect was observed in both cell culture and isolated mitochondria, but not in tissue homogenates. We observed that PEMF stimulates state 3 respiration, which is linked to ATP synthesis. The fact that predominantly ATP synthesis-linked respiration is affected, suggests that PEMF impacts specific ATP synthesis component, which is not required for respiration induced by increased permeability of the IMM. Since mitochondrial ETC is involved in both types of respiration we assume that the PEMF facilitates the process of ATP synthesis. The latter can be due to elevated enzymatic activity of ATP-synthase or facilitation of ADP delivery to this enzyme. PEMF can theoretically affect enzymatic activity via radical pair mechanism, based on transition between singlet and triplet forms of protein based uncoupled electrons^22^. The most important transporter of ADP is adenine nucleotide translocator (ANT) in the IMM. However, this transporter is not regulated by IMM potential and consequently it is unlikely to be affected by PEMF.

In contrast, ADP, along with ATP, and other mitochondrial substrates are transported across the OMM primarily via VDAC. VDAC behaves as a general diffusion pore for small hydrophilic molecules^23^. Although the OMM is considered to be much more permeable compared to the IMM it has been shown that the OMM permeability regulates coupled respiration^24^.

While the OMM does not generate a large membrane potential like the IMM, it plays a role in mitochondrial function through its permeability via its interaction with the IMM at contact sites, and its response to metabolic activity^25^. The membrane potential of OMM in HeLa cells was estimated as approx. 25 mV^26^. Of note, there is a general lack of knowledge about pathophysiological functions of OMM, they were addressed much less than the (IMM. Only recently the function of OMM attracts more attention and the corresponding methods to examine its potential were developed^27^. The pathophysiological potential of OMM remains to be explored.

PEMF did not affect the potential of the inner mitochondrial membrane, although we observe a slight decrease in IMM potential. We cannot exclude that PEMF slightly influences the IMM potential, but since it is much higher than those on the OMM (180 mV vs. 25 mV) it cannot be substantially changed. Depending on the orientation of a membrane the PEMF can either increase or decrease the OMM potential. If the potential is increased VDAC remain closed and if decreased VDAC will open. The only membrane fraction with decreased potential will contribute to increased permeability. This may explain why the effects in mitochondrial suspension were more pronounced than in the cells. The concentration of mitochondria in suspension is higher than in cells or tissues, consequently the number of mitochondria contributing to the effect of PEMF is higher. Although the effects of PEMF we observed here are most likely originated from the mitochondrial membrane, we cannot exclude that biological effects of PEMF can also be due to its interaction with plasma membrane.

Finally, our findings are in line with recent reports showing that PEMFs accelerate angiogenesis, tentatively by inducing energy metabolism^7,8^. It has been shown that PEMF improves the ability of human individuals to run (running capacity)^28^, which increases energy demand. Improvement in systemic metabolism in the post-surgical PEMF-treated cohort of patients was also associated with activation of the mitochondria^9^, but again the underlying mechanism and primary PEMF targets were not addressed. An interesting report showed that PEMF applied to the skin in the presence of 10 mM 2,4-dinitrophenol uncoupling the oxidative phosphorylation in the mitochondria still provides a limited beneficial effect^29^ suggesting that PEMF targets other than uncoupled respiration mitochondrial function, which is in line with our results.

In conclusion, the results of this study did not find any evidence of deleterious effects of PEMF. In contrast, we have demonstrated a beneficial effect of PEMF on mitochondrial function and collected the evidence that sheds light on the underlying mechanism, which is linked to ATP synthesis, rather than to electron transfer via mitochondrial ETC. We did not obtain any evidence supporting possible beneficial effect of PEMF on the mitochondrial function upon inflammation in terms of activation of mitochondria inhibited by NO.

## Methods

### General statements for experimental design and methods used in this study

Animal experiments were compiled in accordance with the EU Directive “Directive 2010/63/EU of the European Parliament and of the Council of 22 September 2010 on the protection of animals used for scientific purposes”. The collection of organs from control animals was approved by the Institutional Review Board (Tierschutzgremium) of the Ludwig Boltzmann Institute of Traumatology on March 16, 2023.

All experimental protocols used in this study were compiled in accordance with Biomedical Research principles at the Ludwig Boltzmann Institute of Traumatology.

All experimental methods used in this study were compiled in accordance with international standards, editorial-policies of the Scientific reports, and Biomedical Research principles at the Ludwig Boltzmann Institute of Traumatology.

All methods used in this study were in accordance with 10 principles of ARRIVE guidelines (https://arriveguidelines.org). Specifically for this study: single animals were used as experimental units, experimental animals were not randomized, because control and experimental samples were always derived from the same single animal, conduction of the experiments, evaluation of the data and matching data with groups were done by three different persons. Other details are described in “Methods” section and “Figure” legends.

### Animal experiments

The ex-vivo experiments were performed on tissues extracted from Sprague-Dawley rats (390–500 g; 8–12 weeks, males; Janvier Labs, Le Genest-Saint-Isle, France) that were anesthetized by inhalation of 8% Sevoflurane mixture (Vapor 2000, Dräger, Austria) for at least 12 min until a complete loss of paw pinch reflex and immediately decapitated afterwards (DCAP-M, World Precision Instruments, Sarasota, FL, USA). Animals were euthanized by decapitation in full accordance with all rules of the Austrian animal and experimental protection law, which implement European regulations. The responsible animal welfare oversight body was the “Institutional Review Board (Tierschutzgremium) of the Ludwig Boltzmann Institute of Traumatology. Liver (median and left lobes) and muscles (m. gastrocnemius) were extracted immediately after decapitation and kept in ice cold ringer solution. The tissues were homogenized in the preparation buffer (0.25 M saccharose, 10 mM Tris, 0.5 mM EDTA, 5 mg/ml fatty acid-free bovine serum albumin, pH 7.2) in a glas-teflon homogenisator either 1 to 4 w/v for experiments with homogenates or 1 to 20 (w/v) for isolation of mitochondria.

### Cell culture experiments

Cell culture experiments were performed with LHCN-M2 cell line. Cells were growing in the cell culture medium containing Dulbecco’s Modified Eagle’s Medium (DMEM) – high glucose (DMEM) + 10% FCS + 1% Glutamine + 1% Penicillin-Streptomycin. Cells were harvested with 1 million/mL in 15mL polystyrene tubes and seeded in 6 well plates and left at 37 °C overnight in incubator (for fluorescent staining next day) or placed in a glass vessel (3 million/vessel, for determination of mitochondrial respiration. Cells, either in plates or in glass vessels were treated for 10 min at level with PEMF (Hofmag, UK, for details see next section). Immediately after treatment the plates were stained with a corresponding fluorescent dye and moved to the microscope (LSM 510, Zeiss), while glass vessels were treated with 12.2 µM digitonin and moved to the high-resolution respirometer (OROBOROS Oxygraph-2k) to determine respiration linked to ATP synthesis with different mitochondrial substrates as well as total capacity of respiratory chain (see below).

### Pulsed electromagnetic field

As newly developed PEMF source using a parallel resonant circuit, in which the semiconductor switch is excluded from the oscillating pathway was used. This configuration eliminates the high resistance losses characteristic of conventional devices, thereby prolonging oscillation decay times to approximately 1 ms—an order of magnitude longer than standard PEMF systems. As a result, generated pulse delivers substantially greater energy over time as quantified as an increased area under the sinusoidal curve. These long duty cycle of 1 ms/sec paired with a low input energy were used to provide an efficient sinus shaped PEMF signal (also dubbed “Synu Field” by the manufacturer Hofmeir Magnetics Limited, Reading, UK). The PEMF Applicator was used in combination with its small loop with an outer diameter of 18 cm. The magnetic field was measured using a from the manufacturer calibrated 3D magnetic field probe (Projekt Elektronik, Berlin, Germany) to determine the properties of the magnetic field. The 2 ms long pulse train, in the shape of a dampened sine wave with a field frequency of 30 kHz and a repetition frequency of 8 Hz. Its maximum magnetic flux density was measured close to the coil, resulting in a peak-to-peak value of 77 mT. An overview of the measured properties can be found in Table 2, a typical PEMF wave is shown in the Fig. S5.

**Table 2:** Measured magnetic field properties of the used PEMF device.

Magnetic flux density (Peak to Peak)	Field frequency	Repetition frequency	Signal shape
77 mT	30 kHz	8 Hz	Dampened sine wave

### Detection of reactive oxygen nitrogen species and mitochondrial membrane potential

96 well plates were used to determine the levels of ROS, NO and mitochondrial membrane potential (MMP). We have determined cytoplasmic reactive oxygen and nitrogen species. Cytoplasmic ROS were determined by staining cells with 20 µM, 2’,7’-dichlorodihydrofluorescein diacetate (DCF-DA, ex/em: 488/505 nm), The relative concentration of NO was determined by staining with 10 µM 4-Amino-5-Methylamino-2’,7’-Difluorofluorescein Diacetate (DAF-FM diacetate, ex/em: 488/505 nm). Stains were performed according to the manufacturer’s manual. All fluorescent dyes were obtained from Invitrogen. Imaging was recorded with an inverted confocal microscope (LSM 510 Zeiss), and the Image analysis was performed with either AxioVision (Version 3.2; Carl Zeiss) or Image J (National Institute of Health). Detection of mitochondrial membrane potential. Mitochondrial membrane potential was determined after staining with 0.5 µM of Tetramethylrhodamine methyl ester (TMRM, ex/em: 552/574nm). The cells were incubated with either DCF-DA, DAF-FM or TMRM for 30 min at 37 °C.

### Isolation of mitochondria

Rat liver mitochondria (RLM) were isolated from adult male Sprague–Dawley rats as described below. Rat liver (median and left lobes) was extracted, washed, minced and homogenized (1 to 20 w/v, in a Glass/Teflon Potter) in the preparation buffer. The homogenate was centrifuged at 800 g and 4 °C for 10 min; the supernatant was subsequently centrifuged at 12 000 g for 10 min. The resulting pellet was resuspended in fresh buffer and again centrifuged at 12 000 g for 10 min. Isolated mitochondria were resuspended in preparation buffer and stored on ice for the duration of the experiment. Protein concentration in mitochondrial suspension was determined by Cobas using the firm KIT and adjusted to 40 mg protein/ml. Mitochondrial suspension was exposed to PEMF for 5 min and then moved to the high-resolution respirometer. In the detector chamber mitochondria were diluted to 1 mg protein/ml.

### Determination of mitochondrial respiratory function

Mitochondrial respiratory activity was measured with high-resolution oxygraphy (Oroboros O2k, Oroboros Instruments, Innsbruck, Austria) and data was recorded using the Oroboros DatLab software (Oroboros Instruments, Innsbruck, Austria). The protocol was performed as previously described (Weidinger et al., 2023). To initiate mitochondrial respiration, we added different mitochondrial substrates to the end concentration of 10 mM. Next, we stimulated state III respiration and ATP synthesis by adding ADP (1 mM) to each chamber. These steps allowed us to estimate the contribution of the complex I- linked respiration. To dissect the contribution of complex II, a potent complex I inhibitor rotenone (1.4 mM) was added. Subsequently, a complex II substrate succinate (10 mM) was introduced, thus now driving the respiration exclusively through complex II. Next, maximal respiratory capacity of the ETC was measured after stepwise titration with the uncoupler FCCP (0.3 µM). Finally, mitochondrial respiration was terminated by the addition of complex III inhibitor Myxothiazol (12.5 µM). All reagents were obtained from Sigma-Aldrich (Vienna, Austria). Several samples were exposed to the blue light directly in the chamber of high-resolution oxygraph via the front window. The blue LED (470 nm) was manufactured by REPULS Lichtmedizintechnik GmbH, Austria.

**Fig. 6:**
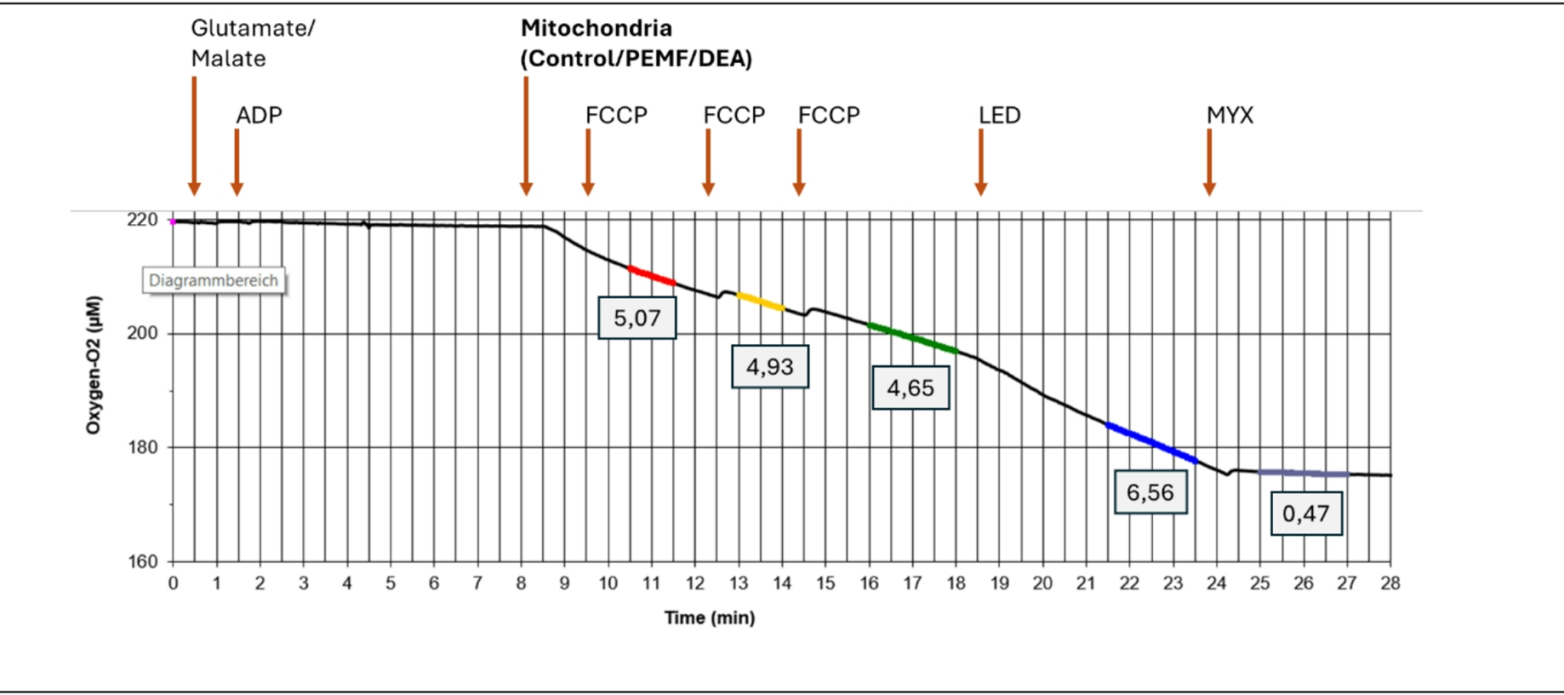
Example of calculating the mitochondrial respiration rate and the recovery effect of blue light. The segments selected for slope calculations are highlighted in different colors. The oxygen flux after myxothiazol treatment was subtracted from raw values. ADP –adenosine diphosphate; FCCP – carbonyl cyanide-4-(trifluoromethoxy)phenylhydrazone; LED – blue light source; Myx – myxothiazol; PEMF– pulsed electromagnetic fields; DAA – diacetyl-adenosine; DEA-NONOate – a nitric oxide (NO) donor.

Unless otherwise specified in the figure, respiration rate calculations were performed as shown in Fig. 6. Figure 6 illustrates a typical oxygen uptake trace from the chamber containing mitochondria treated with DEANONOate and the irradiation with the blue light. After each treatment, the linear portion of the oxygen consumption trace was selected (highlighted in different colours), and the slope of this segment was determined. Calculations were performed using self-made Macro in EXCEL (Microsoft).

### Treatment with DEA-NONOate

10 mM DEA-NONOate was dissolved im 10 mM KOH. The control samples became corresponding amount of KOH. To keep pH the upon addition of KOH we used 20 mM TRIS buffer instead of 10 mM. The OROBOROS champers were filled with substrate and ADP, then the treated RLM were added and then FCCP to induce uncoupled respiration. After following addition of FCCP did not change the rate of respiration, the samples were exposed to blue light. Protein in homogenate is approx. 20 mg P/ml, while the mitochondrial suspension was 35 mg P/ml. Because of this we increased the concentration of DEA-NONOate from 250 µM to 500 µM.

### Determination of nitric oxide release

The NO levels in the medium were measured with Sievers nitric oxide NOA-280i analyzer (NOA). The major product of NO oxidation in the medium is nitrite (NO_2_^−^). A reducing agent (NaI) is used to convert and measure nitrite back to NO via the following reaction:

$${{\mathrm{I}}^ - }+{\mathrm{N}}{{\mathrm{O}}_{\mathrm{2}}}^{ - }+{\mathrm{2}}{{\mathrm{H}}^+} \to {\mathrm{NO}}+0.{\mathrm{5}}{{\mathrm{I}}_{\mathrm{2}}}+{{\mathrm{H}}_{\mathrm{2}}}{\mathrm{O}}$$

Afterwards, the NO was quantified based on the chemiluminescent reaction with ozone. The quantification was performed by determination of the areas under the chemiluminescence peaks or under the steady state part chemiluminescence kinetics for a given time.

### Statistical analysis

All data are presented as mean ± standard error of mean (SEM). Comparisons between groups were calculated by ANOVA followed by Holm-Šídák post Hoc multiple comparison test or LSD test if each comparison stands alone (Figs. 1 and 2). If the p values < 0.05 the differences were considered as statistically significant. All calculations were performed using GraphPad software (GraphPad Software, Inc., San Diego, CA).

## Supplementary Information

Below is the link to the electronic supplementary material.

Supplementary Material 1

## Data Availability

All data files will be available from the digital data repository for published research of the Ludwig Boltzmann Society (https://creed.lbg.ac.at).
