# Supplementary material for: Interaction of pulsed low frequency electromagnetic field (PEMF) with mitochondria
Source: Sci Rep. 2026 Jan 30;16:6681. doi: 10.1038/s41598-026-37527-6 (PMC12914018; doi:10.1038/s41598-026-37527-6)
Supplement: Supplementary file 1 — Supplementary Material 1 [file 41598_2026_37527_MOESM1_ESM.pdf]

Supplementary Material

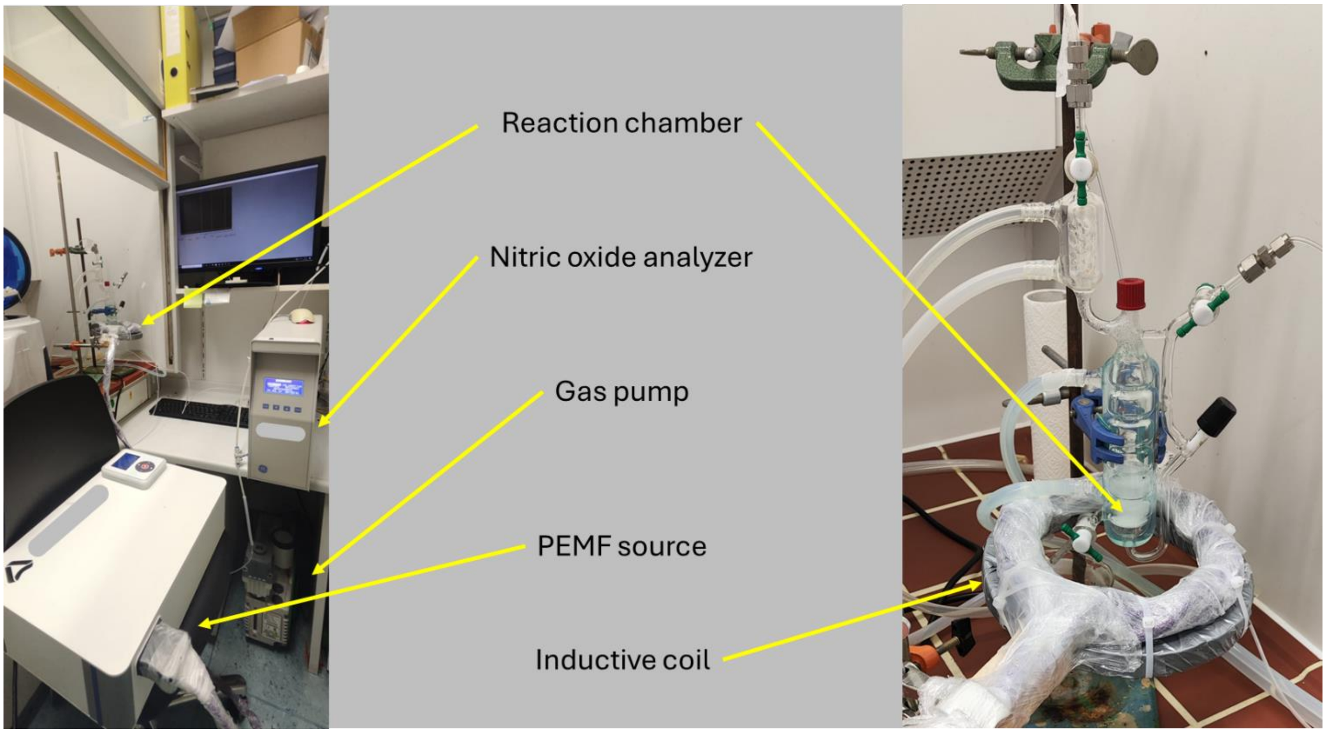

Figure S1. Chamber connected simultaneously to PEMF generator and NOA. NO-donor was injected into the chamber through septum fixed with the red adaptor on the top of the chamber. The chamber was flashed with nitrogen gas. The nitrogen flow was directed to NOA. The chamber was placed into the electromagnetic loop. The nitrogen flow was used to avoid the reaction of NO with oxygen. DEA NONOate, a NO-donor with approx. 15 min half-life was selected for these experiments. This NO donor is stable at alkali pH, consequently the control samples received corresponding amount of KOH. Small volumes of KOH and NO-donor did not change the pH of the buffer used.

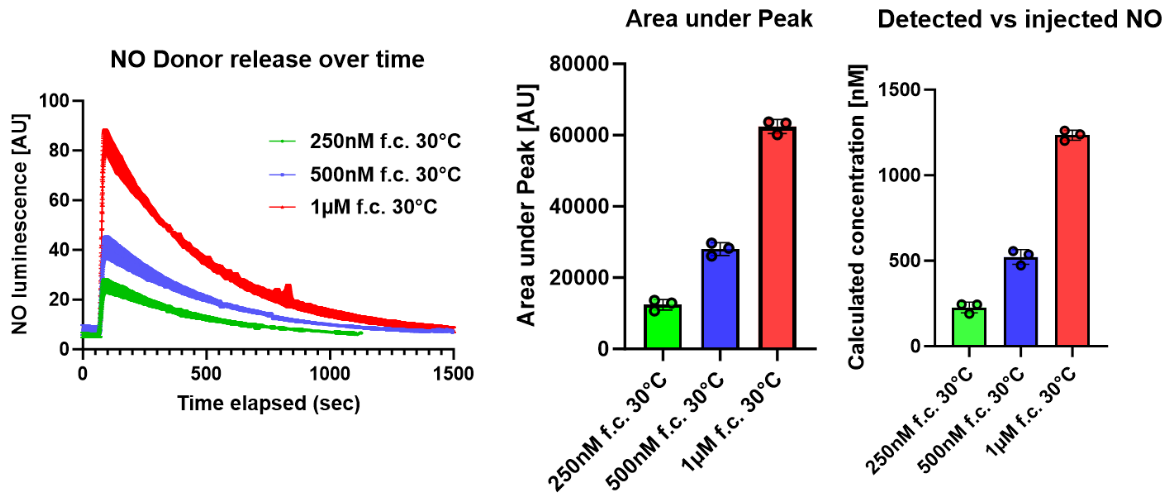

Figure S2: Effect of DEA NONOate concentration on the time courses of NO release at fixed temperature (30C). **A.** Kinetics of NO release; data is shown as mean ±SD, n=3. **B.** Area under the kinetics curve. **C.** Corresponding concentrations of NO.

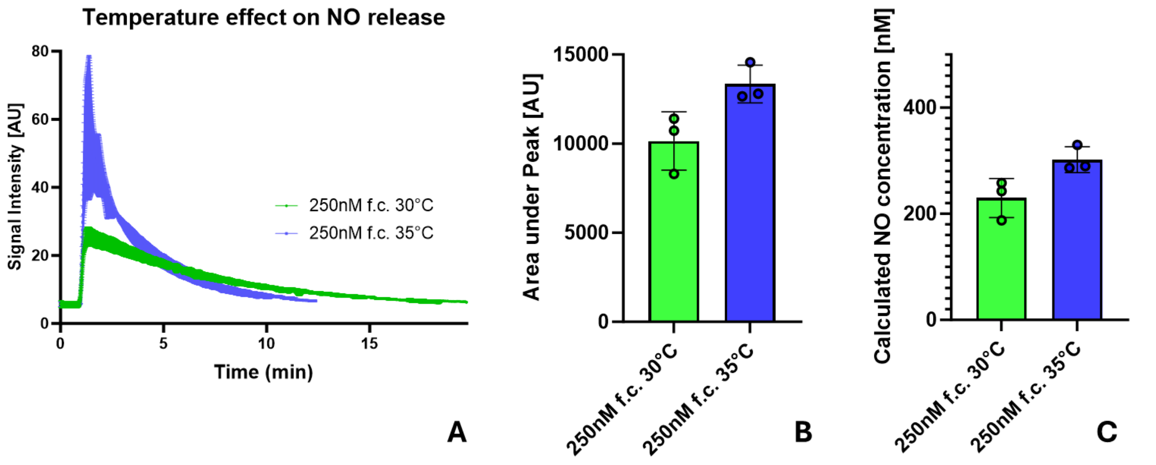

Figure S3. Effect of the temperature on the NO release from DEA-NONOate. Experimental conditions: 250 nM of NONOate was added to the reaction chamber at 30 and 35C. ***A****. traces of NO release. Data are shown as mean ±SD, n=3.* ***B.*** *Area under the kinetic curves vs. added concentration of NO-donor.* ***C.*** *Added concentration of NO-donor vs. calculated concentrations of NO. The data show that we have a reproducible NO release in the experimental model.*

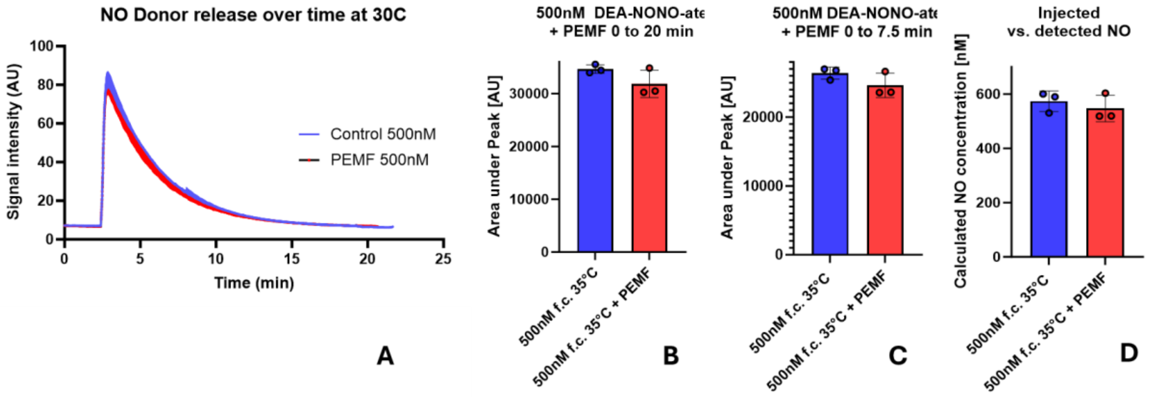

*Figure S4. Effect of the PEMF on time course of NO release from 500 nM of DEA-NONO-ate. **A**. time course; **B** quantifications. F.c. 500nM, 35C, NOA measurement Pressure: 5.5 Torr. **A**. traces of NO release. Data are shown as mean ±SD, n=3. **B**. Area under the kinetic curves vs. added concentration of NO-donor calculated within 20 min. **C**. Area under the kinetic curves vs. added concentration of NO-donor calculated within 7.5 min. **D.** Added concentration of NO-donor vs. calculated concentrations of NO. The data show that we have a reproducible NO release in the experimental model and the kinetics of NO release is not affected by PEMF. This ensures that if any change appears in tested biological models it is not due to modulation of NO-release from NO-donor by PEMF. The data were analyzed by one-way ANOVA followed by Holm-Sidak's multiple comparisons test. The data are presented as mean ± SEM (error bars), experimental points (n=) are shown in the corresponding bars. We did not observe statistically significant improvement of the respiration.*

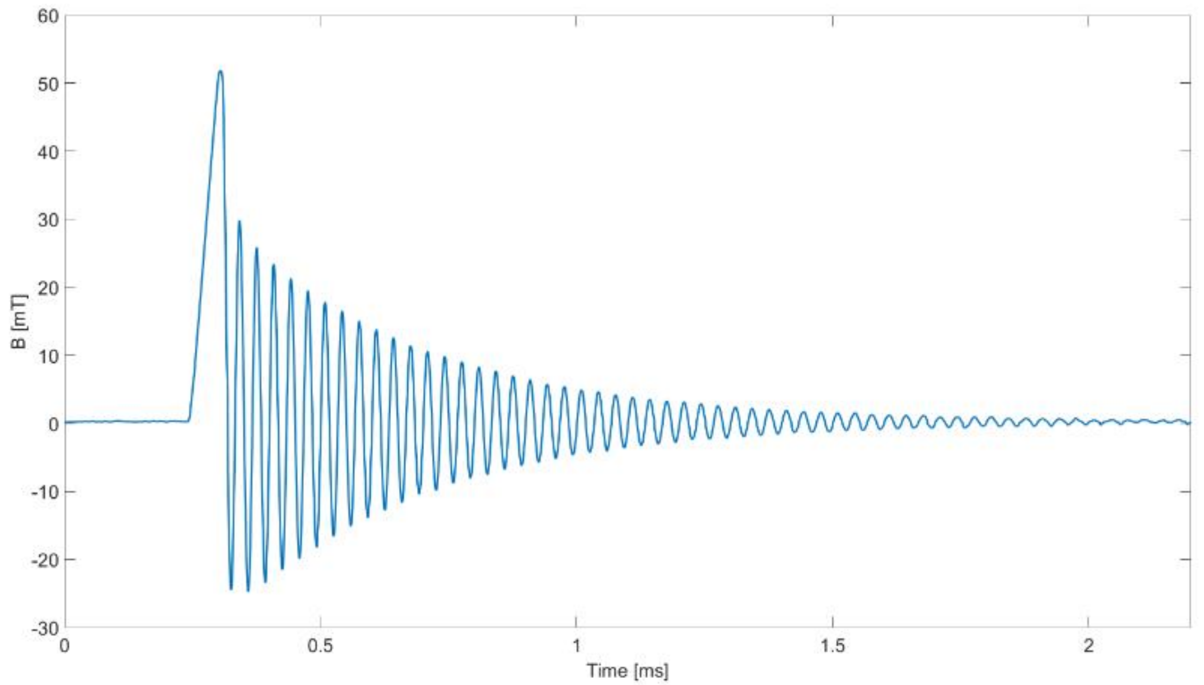

Figure S5. Single Pulse Train of the used PEMF device. The 2 ms long pulse train, in the shape of a dampened sine wave with a field frequency of 30khz and a repetition frequency of 8Hz.
